# Importance of Infection Control in Anesthesiology

**DOI:** 10.7759/cureus.97066

**Published:** 2025-11-17

**Authors:** Jason Jogie, Joshua A Jogie

**Affiliations:** 1 Intensive Care Unit, Port-of-Spain General Hospital, Port-of-Spain, TTO; 2 Faculty of Medical Sciences, The University of the West Indies, St. Augustine, TTO

**Keywords:** anesthesiology, central venous catheters (cvcs), hand hygiene, infection control, perioperative safety

## Abstract

Infection control plays a critical role in anesthesia. Anesthesia often involves close contact with patients and invasive procedures. Anesthesiologists care for patients before, during, and after surgery. This review explains why infection prevention is important. It looks at steps like patient decolonization, hand washing, proper care of vascular access sites, and cleaning the environment. It also suggests ways to improve infection control and urges anesthesiologists to follow evidence-based guidelines. This includes managing external lines, like Central Venous Catheters (CVCs), to lower infection risks. The review shows that following infection prevention protocols leads to better patient outcomes. The recommendations come from groups like the Centers for Disease Control and Prevention (CDC) and the Society for Healthcare Epidemiology of America (SHEA). These are based on randomized controlled trials and expert agreement. The goal is to fill gaps in what healthcare professionals know and what they do in practice. By doing so, we can improve patient safety and quality of care through better infection control in anesthesiology.

## Introduction and background

Preventing infections related to surgery and invasive medical procedures depends on several steps that work together. These steps include cleaning a patient’s skin before an operation, ensuring proper hand hygiene among healthcare workers, maintaining clean intravenous (IV) lines, and keeping the entire environment clean. Each measure alone can help reduce infection risk. However, research shows that using all these measures together is more effective than using any single step alone. In other words, a bundle of evidence-based steps offers a stronger defense against infections [1].

When experts talk about preventing device-related and surgical site infections, they often focus on a few key behaviors. These behaviors include washing hands at the correct times, wearing protective equipment such as masks and gloves, using proper sterile barriers when placing lines, and disinfecting surfaces where germs might settle. Such activities aim to block the chain of infection [1]. When healthcare workers follow these guidelines consistently, the rate of infections can drop, and patients benefit from safer, cleaner care [1].

Hand hygiene is one of the simplest and most important ways to stop germs from spreading [2]. Yet many healthcare workers struggle to maintain perfect hand hygiene practices. They might lack easy access to handwashing stations or alcohol-based hand rubs. Their workload might be high, leaving them little time to clean their hands as often as guidelines suggest. They may find that certain hand hygiene products irritate their skin, causing dryness or discomfort [2]. In anesthesia, this problem can be even more complex. Anesthesia providers move quickly, adjust medications, handle equipment, and manage patients’ airways in real time. The constant shift of tasks can make regular hand cleaning feel difficult [2]. Experts suggest finding practical ways to ensure that anesthesia providers and all operating room staff can clean their hands more often and more easily [2].

The operating room (OR) is a high-risk environment for infections. Many procedures happen at once. Different staff members do many tasks. The patient may have multiple devices placed, including central venous catheters (CVCs) and other invasive lines [2]. These devices offer helpful ways to give fluids, medications, and measure vital signs. However, they also create opportunities for germs to enter the bloodstream if not managed with strict infection control steps. Studies have shown that anesthesia providers do not always follow the same infection prevention rules as surgical staff. This may be because anesthesia providers work in a zone that feels separate from the sterile surgical field. Sometimes, there is not enough oversight, and the OR team may not clean surfaces or equipment thoroughly between patients [2]. This inconsistent adherence shows the need to focus on improving infection control methods for all staff in the OR. Better hand hygiene and consistent use of barriers, such as gloves and sterile drapes, can help [2].

This review will focus specifically on managing external lines, such as CVCs, that often remain in place for long periods. These lines are common in the intensive care unit (ICU) and in other settings where patients need close monitoring or long-term treatments. While these lines are valuable tools, they also carry an increased risk of central line-associated bloodstream infections (CLABSIs). CLABSIs can lead to longer hospital stays, higher costs, and worse outcomes for patients. By understanding when and how to change these lines, what disinfectants to use on the skin, and how to maintain them, we can lower the risk of CLABSIs and improve patient safety.

A key recommendation for preventing infections when placing a CVC is to use full sterile barriers. This includes wearing a mask, cap, sterile gown, and sterile gloves. It also means placing a large sterile drape over the patient’s body where the line will be inserted. These measures help keep the insertion site free of germs. Guidelines also suggest using antiseptics like alcohol-based chlorhexidine solutions (with more than 0.5% chlorhexidine in alcohol) to clean the patient’s skin before placing the line [3,4]. Studies support the idea that using chlorhexidine skin preparations lowers the risk of line infections. Another established best practice is choosing the best vein for catheter placement. Evidence suggests that using the subclavian vein for CVCs in adult ICU patients is associated with fewer infections than other sites, such as the femoral vein [3,4].

After the line is in place, there are other strategies to reduce infection risk. For example, using dressings that contain chlorhexidine can help prevent germs from growing at the insertion site. Using securement devices that do not require sutures can also reduce infection risk by avoiding extra breaks in the skin. Another recommendation is to change IV sets that do not contain blood or lipid solutions every seven days. This practice lowers the chances that contaminants will build up in the tubing and then enter the bloodstream [3].

Preventing CLABSIs in hospitals is a major priority. Patients in ICUs face high risks for these infections because they often need multiple devices and long-term care. Each CLABSI can mean more days in the hospital and more interventions, which cost both time and money. Beyond the financial impact, each infection can harm patients and delay their recovery. Understanding what causes CLABSIs makes it possible to put better prevention steps in place. Better prevention steps save lives, reduce suffering, and improve how healthcare systems function [3].

The tips and rules that guide infection prevention efforts come from a mix of high-quality research and expert opinions. Agencies around the world have developed guidelines based on evidence. For example, groups like the Centers for Disease Control and Prevention (CDC) in the United States or similar organizations in other countries review current studies, run trials, and consult experts. They then produce recommendations that healthcare providers can follow. These recommendations change over time as new evidence emerges. They encourage hospitals to adopt practices known to reduce infections and update those practices when better information becomes available [3,4].

However, despite having guidelines, there are still gaps between what we know and what we do. Not all hospitals or care teams follow the same rules. Some healthcare workers may not know the latest recommendations, while others may lack the resources to follow them. There might be disagreement over the best antiseptic to use or how frequently to change certain devices. This variation leads to uneven results and makes it harder to ensure that all patients receive the same level of protection [3].

Different groups, such as The Joint Commission in the United States, offer different sets of guidelines. As a result, one hospital might follow one set of rules, while another hospital follows a different set. Everyone agrees on some basics, like using sterile barriers and cleaning the insertion site with an antiseptic solution, but recommendations for line maintenance, dressing choice, or when to change IV sets can differ. These differences can cause confusion and make it challenging to compare results across hospitals and regions [4,5].

The way we manage CVCs and other lines affects patient outcomes. When guidelines are clear and consistently followed, CLABSIs drop. When practice is inconsistent, patients face higher risks. Reducing CLABSIs matters because it improves patient safety. It also reduces healthcare costs, hospital length of stay, and the use of antibiotics. Over time, lowering infection rates can improve trust in healthcare systems and show that the facility is committed to patient well-being [3,4].

But to achieve these improvements, we need to close knowledge gaps. Researchers must find better ways to standardize guidelines so that everyone in every hospital understands what to do and why. Education and training are important. Healthcare workers need to know why certain steps matter, how to perform them correctly, and what evidence supports them. Regular assessments and feedback can help ensure that staff follow best practices. Local leadership must also support these measures by providing enough supplies and enough time for proper line care.

This review aims to gather evidence and highlight best practices for preventing CLABSIs through better management of CVCs and other invasive devices. By understanding the current guidelines, examining the barriers to following them, and looking for areas where standards differ, we can identify opportunities for improvement. The goal is to produce a set of clear recommendations that hospitals can adopt, making care safer and more consistent.

With improved standardization and better adherence to evidence-based practices, patient outcomes can improve. Fewer infections mean fewer complications, fewer days in the hospital, and a better overall experience for patients and their families. There are challenges in achieving these goals. However, with coordinated efforts, ongoing research, and a willingness to adopt proven methods, we can reduce CLABSIs and improve the quality of care provided to patients around the world.

## Review

When placing central lines, strict aseptic technique reduces the risk of bloodstream infections. This involves wearing a mask, cap, sterile gown, and sterile gloves. It also includes covering the patient with a large sterile drape. Such precautions create a barrier between the healthcare worker’s hands, equipment, and the patient’s open insertion site. If no organisms enter the bloodstream at the time of insertion, the chance of an infection later is lower. Using chlorhexidine-based antiseptic solutions on the patient’s skin before placing the catheter is more effective at killing germs than povidone-iodine or alcohol alone [3,4]. Chlorhexidine works well because it binds to the skin and keeps killing microbes for a longer time. This helps maintain a clean insertion area and can lower the overall infection risk. By following these steps, the initial placement of a central line becomes safer and less likely to lead to complications.

Selecting the best vein for a central catheter matters. In patients who do not need dialysis, choosing the subclavian vein is often linked to fewer infections and fewer mechanical problems than other sites. The subclavian site, although not perfect, tends to have lower bacterial colonization compared to the femoral site. Using ultrasound guidance when placing the line helps the person inserting the catheter see where they are going. This reduces the number of attempts needed to get the line in place, lowering the chance of damaging blood vessels and reducing the need for repeated punctures. Fewer punctures mean fewer entry points for germs. Over time, this reduces the risk of line-related infections [3].

Once the catheter is in place, proper maintenance is essential. It is important to check the site often. Healthcare workers should look for signs of redness, drainage, or pain that might suggest infection. They should also consider if the catheter is still needed. Removing a line that is no longer essential reduces exposure time and infection risk. Dressing changes must be done with care, using sterile technique. If the dressing looks dirty, wet, or loose, it should be changed right away. Previously, some guidelines advised replacing central lines every seven days no matter what. Current recommendations, however, say there is no need to change a well-functioning line on a strict schedule. Only change it if there is a specific reason, such as signs of infection or malfunction. Also, administration sets that do not carry blood, blood products, or lipid-based solutions should be changed at least every 96 hours to keep contamination low [4]. An administration set for an IV lipid fat emulsion should be replaced every 24 hours. This recommendation applies to tubing that is used to administer blood, blood products, or fat emulsions (whether combined with other fluids or infused separately) [6].

Some patients face higher infection risks than others. In such cases, using catheters that have antimicrobial or antiseptic coatings can help. These coatings release small amounts of agents that kill or slow the growth of bacteria and fungi. Studies show that these special catheters can reduce bloodstream infection risk, especially when the line remains in place for more than five days [3]. This is helpful in environments where infection rates are hard to control, or where patients have conditions that make them more vulnerable.

Dressings that contain chlorhexidine can also provide continuous protection at the insertion site. These dressings slowly release antiseptics onto the skin surface. By doing this, they help prevent the growth of bacteria around the catheter site. Research shows that chlorhexidine-impregnated dressings lower CLABSIs and are considered an important element of overall catheter care [3].

Proper training of healthcare staff is critical. Everyone involved in inserting and maintaining central lines must know the correct procedures and understand why these steps matter. Ongoing education ensures that staff remain aware of best practices. Competency checks, periodic training sessions, and structured assessments help reinforce skills and update workers on new guidelines [3,4]. When staff are well-trained, patients have fewer infections and better outcomes.

Monitoring infection rates is also important. Healthcare facilities should keep track of CLABSI rates to see if their interventions are working. Regular surveillance includes checking patient records, laboratory results, and other data to identify cases of line-related infection. By comparing current infection rates to previous periods or to other hospitals, healthcare managers can spot trends. They can then adjust their policies, retrain staff, or introduce new measures as needed [3,4]. Over time, these efforts help maintain high standards of care.

Central venous catheters (CVCs) carry a higher infection risk than other types of vascular access devices. Evidence suggests that the infection risk also depends on the placement site. Femoral lines are often associated with higher infection rates than subclavian or internal jugular lines. Ultrasound guidance and a full sterile approach help reduce these complications [5,7]. Each step that reduces unnecessary handling and manipulation can lower the chance that bacteria will enter the bloodstream.

Peripheral intravenous catheters (PIVCs) usually present fewer serious infection risks than CVCs, but they are not risk-free. The longer a PIVC remains in place, the greater the chance of infection. Following guidelines for proper insertion, timely replacement, and careful maintenance of PIVCs can keep their infection rates low [8]. Still, even with these simpler devices, careful attention to cleanliness and routine checks is necessary.

Arterial lines also pose infection risks, though generally lower than those of CVCs. Arterial lines allow repeated blood gas analysis and other blood workup. To prevent infections, it is important to use antiseptic-impregnated catheters, maintain good hand hygiene, and handle equipment properly [9]. Even though the risk may be lower than with central lines, it is not zero. Any break in technique can create a pathway for pathogens.

Longer use of any catheter increases infection risk. The reason is simple: the longer a device remains in place, the more time there is for bacteria to grow. Checking daily if the line is still needed is a crucial step. Removing unneeded catheters as soon as possible is a simple but effective method to prevent infections [10].

Preventing line-related infections often involves more than one step. Combining multiple strategies works best. For example, using chlorhexidine for skin disinfection, applying full sterile barriers during insertion, selecting the best site, and following established care bundles can lower infection rates. Training and education help too. When healthcare workers understand why these steps are necessary, they are more likely to follow them every time [11,12].

This knowledge has practical implications. Evidence-based guidelines can improve how we handle lines. By using recommended approaches to insertion, care, and removal, we can improve patient outcomes and lower healthcare costs. Infection prevention requires staying up to date with current research and guidelines.

The risk of infection depends on many factors. Central venous catheters tend to be riskier than peripheral ones. Arterial lines have a risk that falls somewhere in between. Catheter materials, the number of lumens, and whether the catheter has antimicrobial coatings all play a role [13,14]. The patient’s own situation matters too. Patients who are very sick or have weakened immune systems face higher infection risks. Conditions like diabetes and the length of catheter use also increase the chance of infection [10,15].

The clinical setting makes a difference. Intensive care units see more device-related infections because patients there are often very ill and need multiple invasive devices. Even with improvements, these settings remain challenging. Other parts of the hospital can also see infections if staff do not follow proper procedures [11,16].

Insertion and maintenance practices are at the heart of infection control. Poor technique, neglected dressings, and improper site care can lead to infections. Using aseptic methods, changing dressings when needed, and cleaning the catheter hub before each use keep germs out [17,18]. Good hand hygiene and using personal protective equipment (PPE) also help. Staff compliance matters a lot. If workers wash their hands, wear gloves, and follow other rules, infection rates go down [19,20].

Chlorhexidine-alcohol solutions kill a broad range of microbes and help ensure a clean surface before line placement. Letting the solution dry fully before inserting the catheter matters. Even small shortcuts can increase risk. Maintenance protocols like “scrub-the-hub” and using sterile gloves keep contaminants from entering the line [10,11]. Dressings should be changed if they become damp, dirty, or loose. Transparent dressings let healthcare workers see the insertion site without removing the covering, reducing unnecessary exposure.

Education and ongoing competency checks help maintain quality. Training sessions, simulations, and updated protocols keep skills sharp and prevent confusion [15,21,22]. Bundled care interventions, where several proven measures are applied together, have shown success in reducing infections. For example, a bundle might include strict hand hygiene, full barrier precautions, chlorhexidine skin prep, careful site selection, and daily evaluation of line necessity [23,24].

Regarding when to replace lines, studies have shown that routine line changes do not always reduce infections. Instead, replacing a catheter should depend on whether it is still needed or if it shows signs of infection [25,26]. For peripheral lines, some guidelines suggest changing them every 72-96 hours, while others say it is fine to keep them longer if they are still working and clean [27,28].

Some areas remain uncertain. For example, we know antimicrobial-coated catheters can lower infection rates, but we still need more data on their cost-effectiveness and long-term impact on resistance [29,30]. We also need more clarity on the best formulations of chlorhexidine and other antiseptics, and what side effects they might have [31,32].

Future research should continue to compare different approaches. We need to know which dressings, antiseptics, and timing strategies produce the best results over time. Comparative studies can guide more precise recommendations. Past work has shown that chlorhexidine-impregnated sponges help prevent infections [33], but more research can confirm the best overall methods.

Innovations in catheter design may help reduce infections. Better materials, novel antimicrobial coatings, and designs that minimize handling can lower risks. Some studies confirm antimicrobial coatings help [34]. Further improvements might involve new antiseptics or technologies that warn of early infection signs. For example, sensors could alert staff about temperature changes or subtle shifts in line function, allowing for early interventions.

In the end, preventing infections with central lines and other catheters depends on a mix of evidence-based steps and ongoing improvement. By staying informed, following updated guidelines, and paying attention to each detail of insertion and care, healthcare workers can reduce the risk of infection. Future research can fill the gaps in our knowledge, improve designs, and help us find better antiseptics. Over time, these efforts can save lives, reduce complications, and make patient care safer and more reliable.

## Conclusions

Anesthesia professionals have a key role because they work closely with patients before, during, and after surgery. By following strict rules - like washing hands, cleaning surfaces, and handling invasive devices with care - they can help lower the risk of infection. The review shows that a layered approach works best. This means using patient decolonization, steady hand hygiene, careful care of vascular access sites, and proper environmental cleaning. Even though it can be hard to keep up with perfect hand hygiene and follow every rule, the review points out ways to improve. These include making sure everyone follows hand hygiene steps, keeping the environment clean, and using proven methods to manage items like central venous catheters to lower infection rates. The literature in the review gives strong evidence for using these methods in everyday practice. It can help improve patient safety and results. With ongoing education, sticking to good practices, and using a set group of steps, it’s possible to cut down on infections in anesthesiology.
